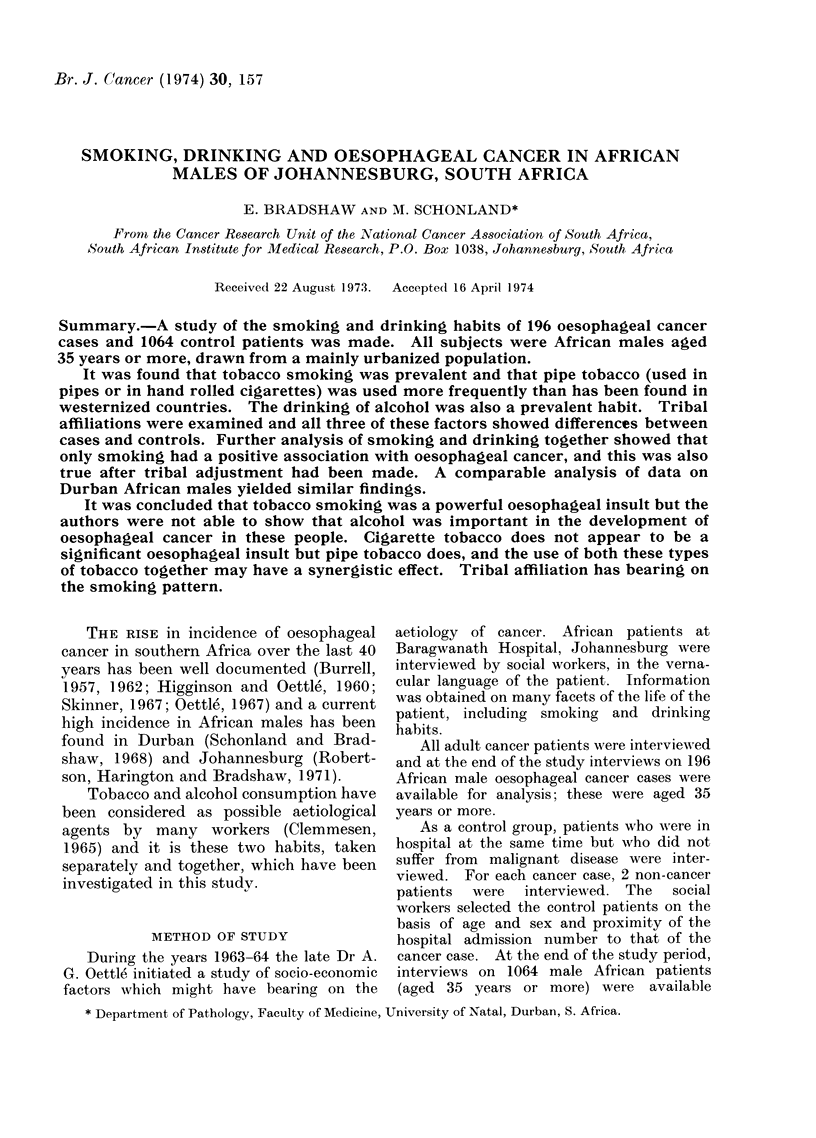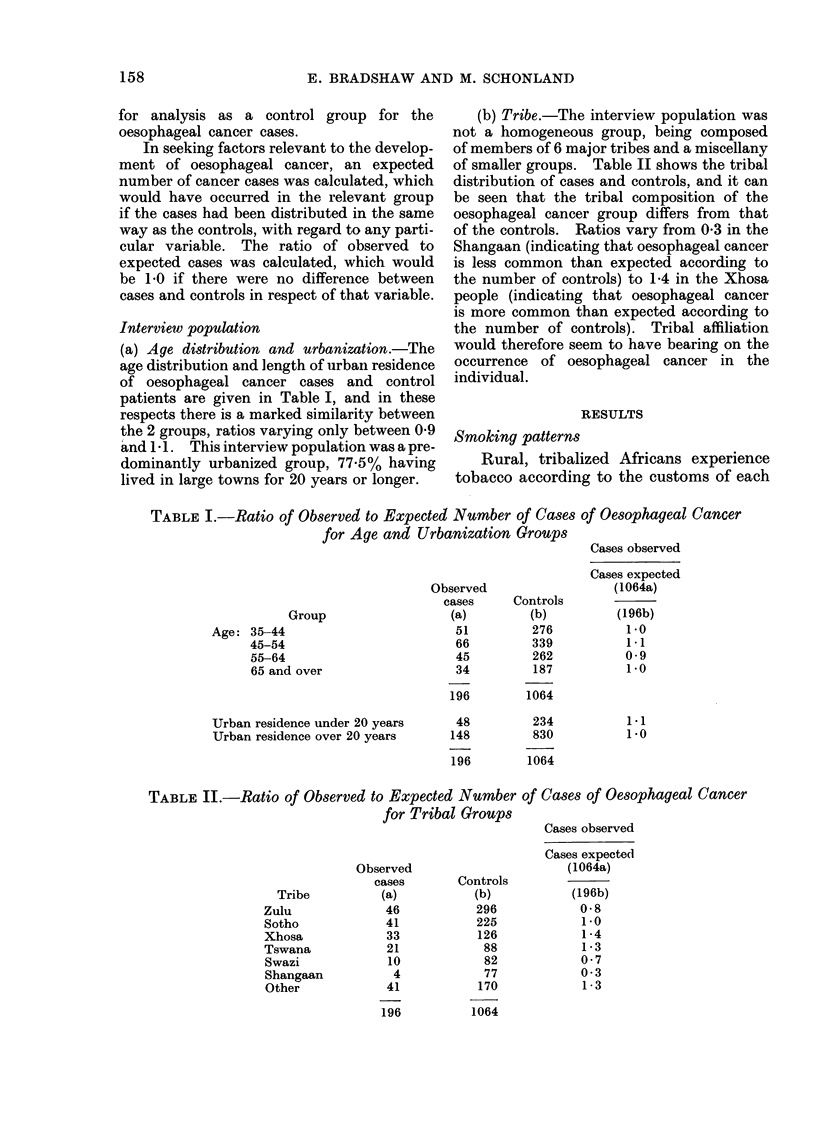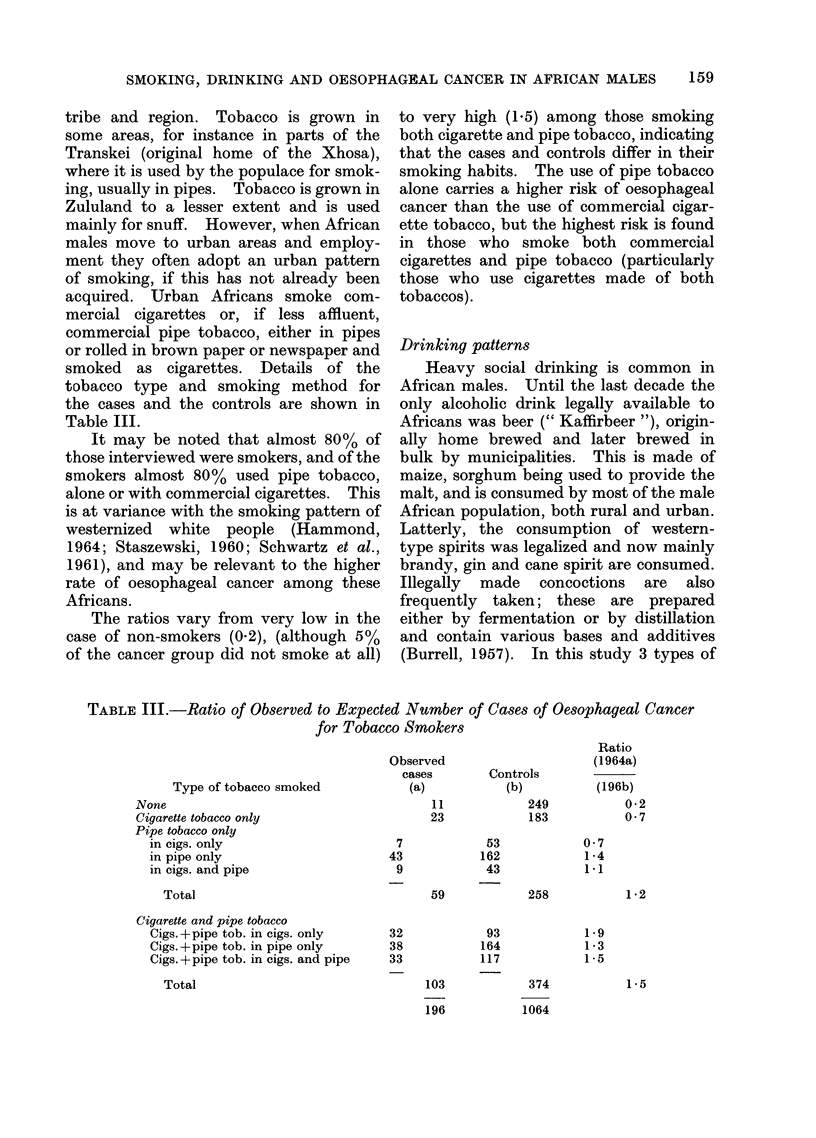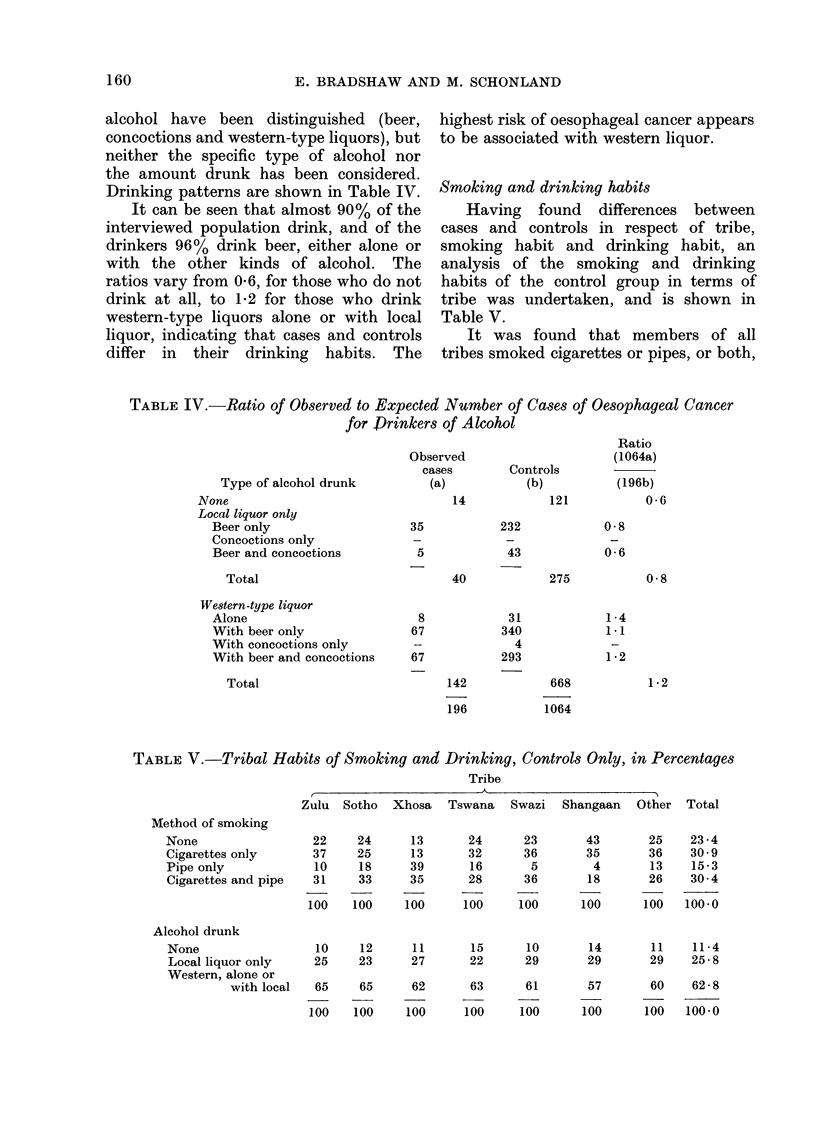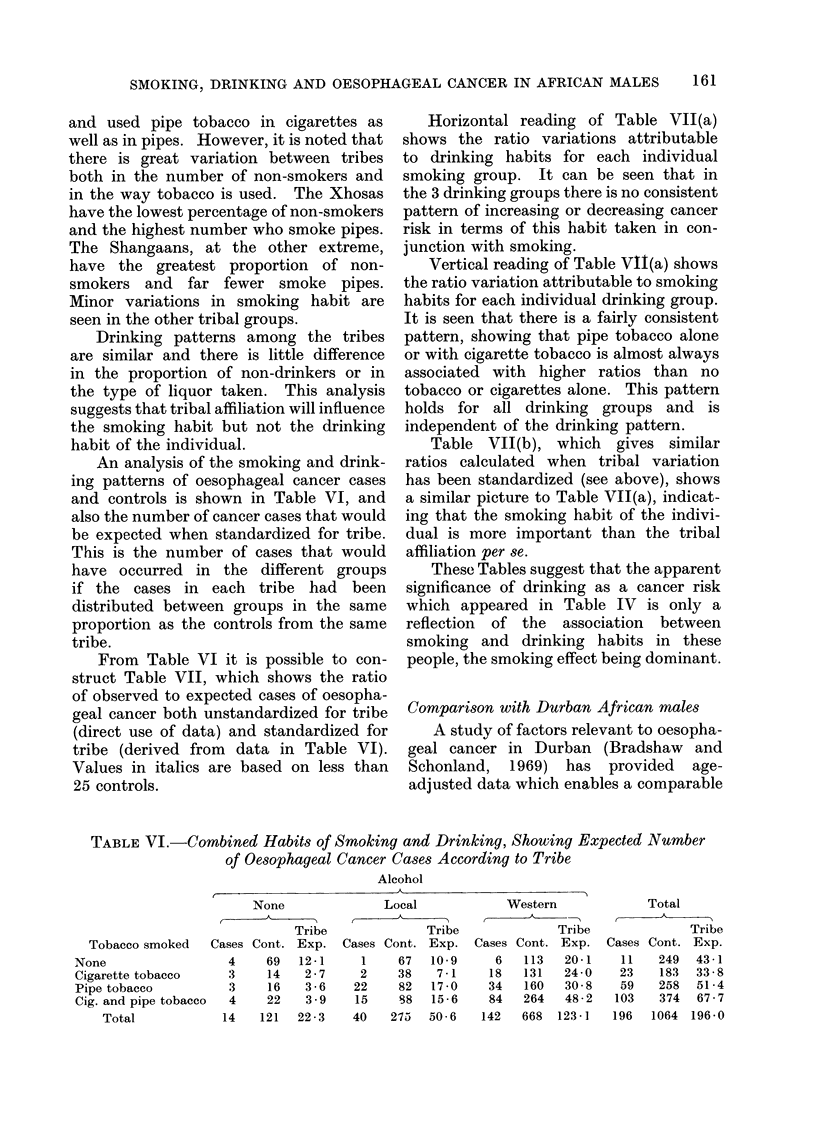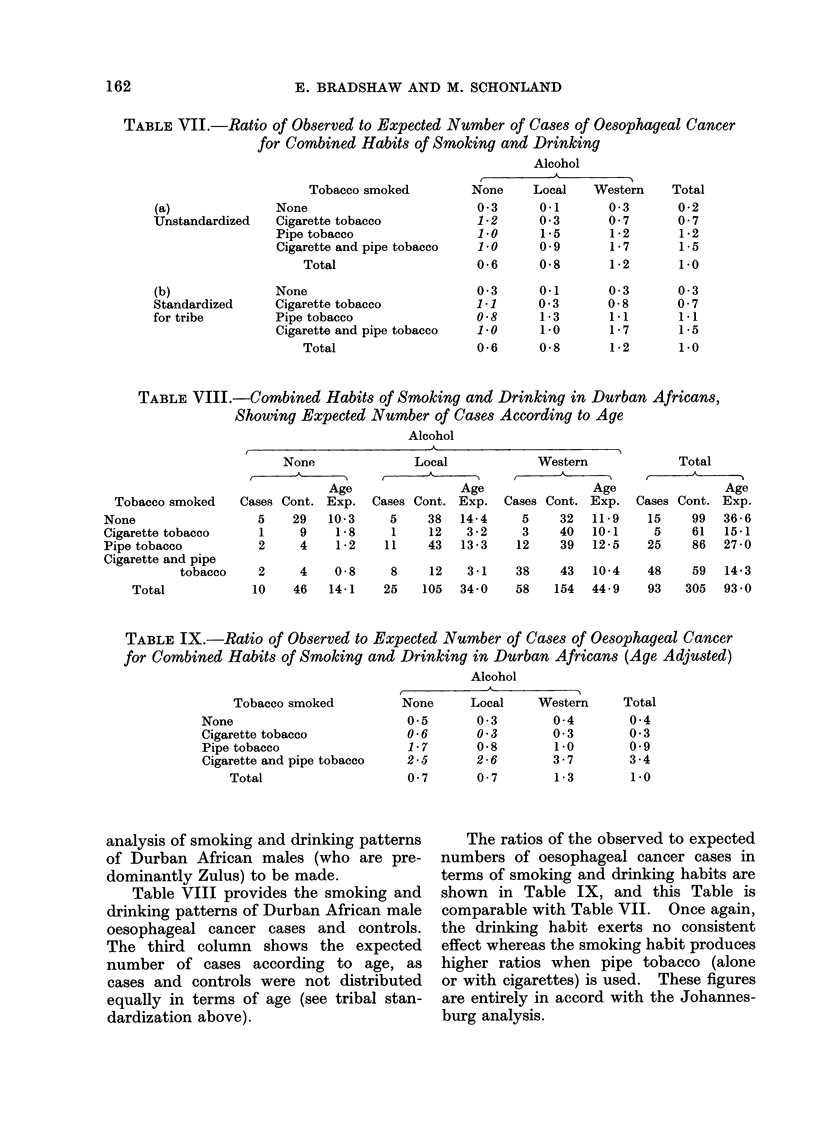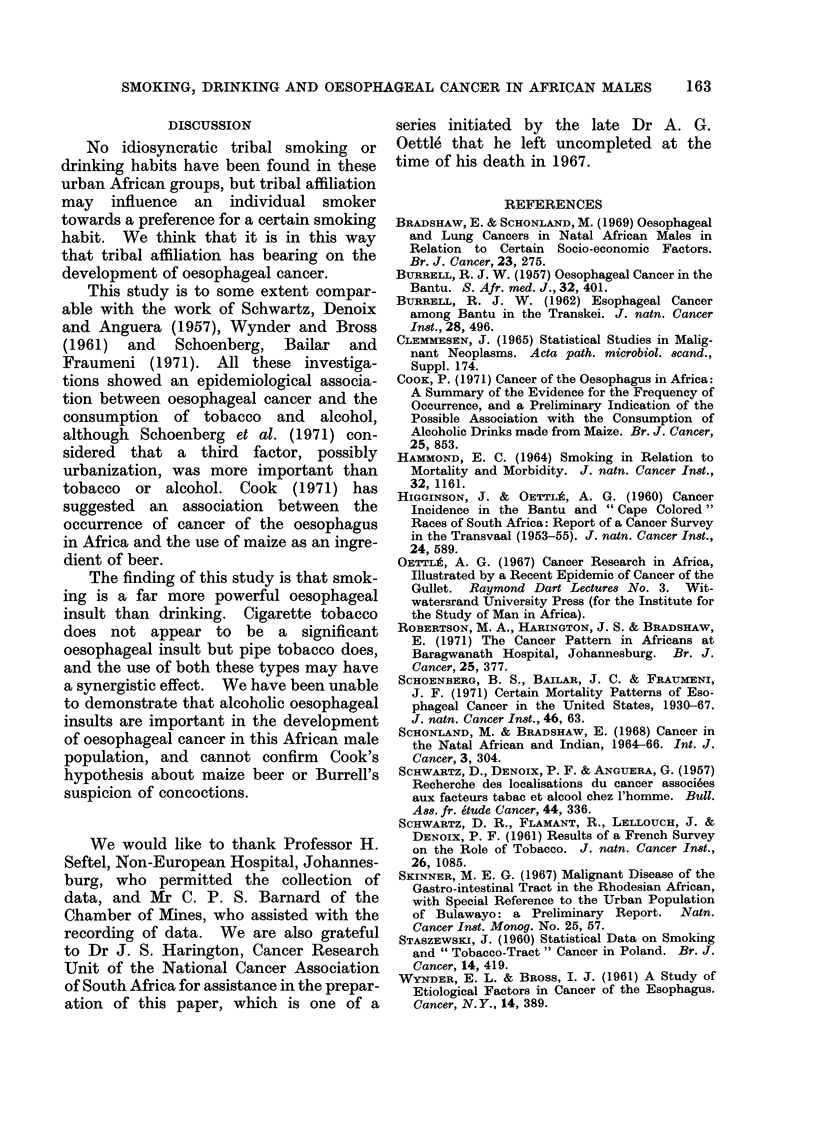# Smoking, Drinking and Oesophageal Cancer in African Males of Johannesburg, South Africa

**DOI:** 10.1038/bjc.1974.127

**Published:** 1974-08

**Authors:** E. Bradshaw, M. Schonland

## Abstract

A study of the smoking and drinking habits of 196 oesophageal cancer cases and 1064 control patients was made. All subjects were African males aged 35 years or more, drawn from a mainly urbanized population.

It was found that tobacco smoking was prevalent and that pipe tobacco (used in pipes or in hand rolled cigarettes) was used more frequently than has been found in westernized countries. The drinking of alcohol was also a prevalent habit. Tribal affiliations were examined and all three of these factors showed differences between cases and controls. Further analysis of smoking and drinking together showed that only smoking had a positive association with oesophageal cancer, and this was also true after tribal adjustment had been made. A comparable analysis of data on Durban African males yielded similar findings.

It was concluded that tobacco smoking was a powerful oesophageal insult but the authors were not able to show that alcohol was important in the development of oesophageal cancer in these people. Cigarette tobacco does not appear to be a significant oesophageal insult but pipe tobacco does, and the use of both these types of tobacco together may have a synergistic effect. Tribal affiliation has bearing on the smoking pattern.


*Br. J. Cancer* (1974) **30**, 157

### SMOKING, DRINKING AND OESOPHAGEAL CANCER IN AFRICAN MALES OF JOHANNESBURG, SOUTH AFRICA

E. BRADSHAW AND M. SCHONLAND*

*From the Cancer Research Unit of the National Cancer Association of South Africa, South African Institute for Medical Research, P.O. Box* 1038, *Johannesburg, South Africa*

Received 22 August 1973. Accepted 16 April 1974

**Summary.—A study of the smoking and drinking habits of 196 oesophageal cancer cases and 1064 control patients was made. All subjects were African males aged 35 years or more, drawn from a mainly urbanized population.**

**It was found that tobacco smoking was prevalent and that pipe tobacco (used in pipes or in hand rolled cigarettes) was used more frequently than has been found in westernized countries. The drinking of alcohol was also a prevalent habit. Tribal affiliations were examined and all three of these factors showed differences between cases and controls. Further analysis of smoking and drinking together showed that only smoking had a positive association with oesophageal cancer, and this was also true after tribal adjustment had been made. A comparable analysis of data on Durban African males yielded similar findings.**

**It was concluded that tobacco smoking was a powerful oesophageal insult but the authors were not able to show that alcohol was important in the development of oesophageal cancer in these people. Cigarette tobacco does not appear to be a significant oesophageal insult but pipe tobacco does, and the use of both these types of tobacco together may have a synergistic effect. Tribal affiliation has bearing on the smoking pattern.**

THE RISE in incidence of oesophageal cancer in southern Africa over the last 40 years has been well documented (Burrell, 1957, 1962; Higginson and Oettlé, 1960; Skinner, 1967; Oettlé, 1967) and a current high incidence in African males has been found in Durban (Schonland and Bradshaw, 1968) and Johannesburg (Robertson, Harington and Bradshaw, 1971).

Tobacco and alcohol consumption have been considered as possible aetiological agents by many workers (Clemmesen, 1965) and it is these two habits, taken separately and together, which have been investigated in this study.

### METHOD OF STUDY

During the years 1963–64 the late Dr A. G. Oettlé initiated a study of socio-economic factors which might have bearing on the aetiology of cancer. African patients at Baragwanath Hospital, Johannesburg were interviewed by social workers, in the vernacular language of the patient. Information was obtained on many facets of the life of the patient, including smoking and drinking habits.

All adult cancer patients were interviewed and at the end of the study interviews on 196 African male oesophageal cancer cases were available for analysis; these were aged 35 years or more.

As a control group, patients who were in hospital at the same time but who did not suffer from malignant disease were interviewed. For each cancer case, 2 non-cancer patients were interviewed. The social workers selected the control patients on the basis of age and sex and proximity of the hospital admission number to that of the cancer case. At the end of the study period, interviews on 1064 male African patients (aged 35 years or more) were available

* Department of Pathology, Faculty of Medicine, University of Natal, Durban, S. Africa.

for analysis as a control group for the oesophageal cancer cases.

In seeking factors relevant to the development of oesophageal cancer, an expected number of cancer cases was calculated, which would have occurred in the relevant group if the cases had been distributed in the same way as the controls, with regard to any particular variable. The ratio of observed to expected cases was calculated, which would be 1·0 if there were no difference between cases and controls in respect of that variable.

*Interview population*

(a) *Age distribution and urbanization.*—The age distribution and length of urban residence of oesophageal cancer cases and control patients are given in Table I, and in these respects there is a marked similarity between the 2 groups, ratios varying only between 0·9 and 1·1. This interview population was a predominantly urbanized group, 77·5% having lived in large towns for 20 years or longer.

(b) *Tribe.*—The interview population was not a homogeneous group, being composed of members of 6 major tribes and a miscellany of smaller groups. Table II shows the tribal distribution of cases and controls, and it can be seen that the tribal composition of the oesophageal cancer group differs from that of the controls. Ratios vary from 0·3 in the Shangaan (indicating that oesophageal cancer is less common than expected according to the number of controls) to 1·4 in the Xhosa people (indicating that oesophageal cancer is more common than expected according to the number of controls). Tribal affiliation would therefore seem to have bearing on the occurrence of oesophageal cancer in the individual.

RESULTS

*Smoking patterns*

Rural, tribalized Africans experience tobacco according to the customs of each

TABLE I.—*Ratio of Observed to Expected Number of Cases of Oesophageal Cancer for Age and Urbanization Groups*

| Group | Observed cases (a) | Controls (b) | Cases observed / Cases expected: (1064a) / (196b) |
|---|---|---|---|
| Age: 35–44 | 51 | 276 | 1·0 |
| 45–54 | 66 | 339 | 1·1 |
| 55–64 | 45 | 262 | 0·9 |
| 65 and over | 34 | 187 | 1·0 |
| | 196 | 1064 | |
| Urban residence under 20 years | 48 | 234 | 1·1 |
| Urban residence over 20 years | 148 | 830 | 1·0 |
| | 196 | 1064 | |

TABLE II.—*Ratio of Observed to Expected Number of Cases of Oesophageal Cancer for Tribal Groups*

| Tribe | Observed cases (a) | Controls (b) | Cases observed / Cases expected: (1064a) / (196b) |
|---|---|---|---|
| Zulu | 46 | 296 | 0·8 |
| Sotho | 41 | 225 | 1·0 |
| Xhosa | 33 | 126 | 1·4 |
| Tswana | 21 | 88 | 1·3 |
| Swazi | 10 | 82 | 0·7 |
| Shangaan | 4 | 77 | 0·3 |
| Other | 41 | 170 | 1·3 |
| | 196 | 1064 | |

tribe and region. Tobacco is grown in some areas, for instance in parts of the Transkei (original home of the Xhosa), where it is used by the populace for smoking, usually in pipes. Tobacco is grown in Zululand to a lesser extent and is used mainly for snuff. However, when African males move to urban areas and employment they often adopt an urban pattern of smoking, if this has not already been acquired. Urban Africans smoke commercial cigarettes or, if less affluent, commercial pipe tobacco, either in pipes or rolled in brown paper or newspaper and smoked as cigarettes. Details of the tobacco type and smoking method for the cases and the controls are shown in Table III.

It may be noted that almost 80% of those interviewed were smokers, and of the smokers almost 80% used pipe tobacco, alone or with commercial cigarettes. This is at variance with the smoking pattern of westernized white people (Hammond, 1964; Staszewski, 1960; Schwartz *et al.*, 1961), and may be relevant to the higher rate of oesophageal cancer among these Africans.

The ratios vary from very low in the case of non-smokers (0·2), (although 5% of the cancer group did not smoke at all) to very high (1·5) among those smoking both cigarette and pipe tobacco, indicating that the cases and controls differ in their smoking habits. The use of pipe tobacco alone carries a higher risk of oesophageal cancer than the use of commercial cigarette tobacco, but the highest risk is found in those who smoke both commercial cigarettes and pipe tobacco (particularly those who use cigarettes made of both tobaccos).

*Drinking patterns*

Heavy social drinking is common in African males. Until the last decade the only alcoholic drink legally available to Africans was beer (" Kaffirbeer "), originally home brewed and later brewed in bulk by municipalities. This is made of maize, sorghum being used to provide the malt, and is consumed by most of the male African population, both rural and urban. Latterly, the consumption of western-type spirits was legalized and now mainly brandy, gin and cane spirit are consumed. Illegally made concoctions are also frequently taken; these are prepared either by fermentation or by distillation and contain various bases and additives (Burrell, 1957). In this study 3 types of

TABLE III.—*Ratio of Observed to Expected Number of Cases of Oesophageal Cancer for Tobacco Smokers*

| Type of tobacco smoked | Observed cases (a) | | Controls (b) | | Ratio (1964a)/(196b) | |
|---|---|---|---|---|---|---|
| *None* | | 11 | | 249 | | 0·2 |
| *Cigarette tobacco only* | | 23 | | 183 | | 0·7 |
| *Pipe tobacco only* | | | | | | |
| in cigs. only | 7 | | 53 | | 0·7 | |
| in pipe only | 43 | | 162 | | 1·4 | |
| in cigs. and pipe | 9 | | 43 | | 1·1 | |
| Total | | 59 | | 258 | | 1·2 |
| *Cigarette and pipe tobacco* | | | | | | |
| Cigs.+pipe tob. in cigs. only | 32 | | 93 | | 1·9 | |
| Cigs.+pipe tob. in pipe only | 38 | | 164 | | 1·3 | |
| Cigs.+pipe tob. in cigs. and pipe | 33 | | 117 | | 1·5 | |
| Total | | 103 | | 374 | | 1·5 |
| | | 196 | | 1064 | | |

alcohol have been distinguished (beer, concoctions and western-type liquors), but neither the specific type of alcohol nor the amount drunk has been considered. Drinking patterns are shown in Table IV.

It can be seen that almost 90% of the interviewed population drink, and of the drinkers 96% drink beer, either alone or with the other kinds of alcohol. The ratios vary from 0·6, for those who do not drink at all, to 1·2 for those who drink western-type liquors alone or with local liquor, indicating that cases and controls differ in their drinking habits. The highest risk of oesophageal cancer appears to be associated with western liquor.

### *Smoking and drinking habits*

Having found differences between cases and controls in respect of tribe, smoking habit and drinking habit, an analysis of the smoking and drinking habits of the control group in terms of tribe was undertaken, and is shown in Table V.

It was found that members of all tribes smoked cigarettes or pipes, or both,

TABLE IV.—*Ratio of Observed to Expected Number of Cases of Oesophageal Cancer for Drinkers of Alcohol*

| Type of alcohol drunk | Observed cases (a) | | Controls (b) | | Ratio (1064a) / (196b) | |
|---|---|---|---|---|---|---|
| *None* | | 14 | | 121 | | 0·6 |
| *Local liquor only* | | | | | | |
| Beer only | 35 | | 232 | | 0·8 | |
| Concoctions only | – | | – | | – | |
| Beer and concoctions | 5 | | 43 | | 0·6 | |
| Total | | 40 | | 275 | | 0·8 |
| *Western-type liquor* | | | | | | |
| Alone | 8 | | 31 | | 1·4 | |
| With beer only | 67 | | 340 | | 1·1 | |
| With concoctions only | – | | 4 | | – | |
| With beer and concoctions | 67 | | 293 | | 1·2 | |
| Total | | 142 | | 668 | | 1·2 |
| | | 196 | | 1064 | | |

TABLE V.—*Tribal Habits of Smoking and Drinking, Controls Only, in Percentages*

| | Tribe | | | | | | | |
|---|---|---|---|---|---|---|---|---|
| | Zulu | Sotho | Xhosa | Tswana | Swazi | Shangaan | Other | Total |
| Method of smoking | | | | | | | | |
| None | 22 | 24 | 13 | 24 | 23 | 43 | 25 | 23·4 |
| Cigarettes only | 37 | 25 | 13 | 32 | 36 | 35 | 36 | 30·9 |
| Pipe only | 10 | 18 | 39 | 16 | 5 | 4 | 13 | 15·3 |
| Cigarettes and pipe | 31 | 33 | 35 | 28 | 36 | 18 | 26 | 30·4 |
| | 100 | 100 | 100 | 100 | 100 | 100 | 100 | 100·0 |
| Alcohol drunk | | | | | | | | |
| None | 10 | 12 | 11 | 15 | 10 | 14 | 11 | 11·4 |
| Local liquor only | 25 | 23 | 27 | 22 | 29 | 29 | 29 | 25·8 |
| Western, alone or with local | 65 | 65 | 62 | 63 | 61 | 57 | 60 | 62·8 |
| | 100 | 100 | 100 | 100 | 100 | 100 | 100 | 100·0 |

and used pipe tobacco in cigarettes as well as in pipes. However, it is noted that there is great variation between tribes both in the number of non-smokers and in the way tobacco is used. The Xhosas have the lowest percentage of non-smokers and the highest number who smoke pipes. The Shangaans, at the other extreme, have the greatest proportion of non-smokers and far fewer smoke pipes. Minor variations in smoking habit are seen in the other tribal groups.

Drinking patterns among the tribes are similar and there is little difference in the proportion of non-drinkers or in the type of liquor taken. This analysis suggests that tribal affiliation will influence the smoking habit but not the drinking habit of the individual.

An analysis of the smoking and drinking patterns of oesophageal cancer cases and controls is shown in Table VI, and also the number of cancer cases that would be expected when standardized for tribe. This is the number of cases that would have occurred in the different groups if the cases in each tribe had been distributed between groups in the same proportion as the controls from the same tribe.

From Table VI it is possible to construct Table VII, which shows the ratio of observed to expected cases of oesophageal cancer both unstandardized for tribe (direct use of data) and standardized for tribe (derived from data in Table VI). Values in italics are based on less than 25 controls.

Horizontal reading of Table VII(a) shows the ratio variations attributable to drinking habits for each individual smoking group. It can be seen that in the 3 drinking groups there is no consistent pattern of increasing or decreasing cancer risk in terms of this habit taken in conjunction with smoking.

Vertical reading of Table VII(a) shows the ratio variation attributable to smoking habits for each individual drinking group. It is seen that there is a fairly consistent pattern, showing that pipe tobacco alone or with cigarette tobacco is almost always associated with higher ratios than no tobacco or cigarettes alone. This pattern holds for all drinking groups and is independent of the drinking pattern.

Table VII(b), which gives similar ratios calculated when tribal variation has been standardized (see above), shows a similar picture to Table VII(a), indicating that the smoking habit of the individual is more important than the tribal affiliation *per se*.

These Tables suggest that the apparent significance of drinking as a cancer risk which appeared in Table IV is only a reflection of the association between smoking and drinking habits in these people, the smoking effect being dominant.

### *Comparison with Durban African males*

A study of factors relevant to oesophageal cancer in Durban (Bradshaw and Schonland, 1969) has provided age-adjusted data which enables a comparable

TABLE VI.—*Combined Habits of Smoking and Drinking, Showing Expected Number of Oesophageal Cancer Cases According to Tribe*

| | Alcohol | | | | | | | | | | | |
|---|---|---|---|---|---|---|---|---|---|---|---|---|
| | None | | | Local | | | Western | | | Total | | |
| Tobacco smoked | Cases | Cont. | Tribe Exp. | Cases | Cont. | Tribe Exp. | Cases | Cont. | Tribe Exp. | Cases | Cont. | Tribe Exp. |
| None | 4 | 69 | 12·1 | 1 | 67 | 10·9 | 6 | 113 | 20·1 | 11 | 249 | 43·1 |
| Cigarette tobacco | 3 | 14 | 2·7 | 2 | 38 | 7·1 | 18 | 131 | 24·0 | 23 | 183 | 33·8 |
| Pipe tobacco | 3 | 16 | 3·6 | 22 | 82 | 17·0 | 34 | 160 | 30·8 | 59 | 258 | 51·4 |
| Cig. and pipe tobacco | 4 | 22 | 3·9 | 15 | 88 | 15·6 | 84 | 264 | 48·2 | 103 | 374 | 67·7 |
| Total | 14 | 121 | 22·3 | 40 | 275 | 50·6 | 142 | 668 | 123·1 | 196 | 1064 | 196·0 |

TABLE VII.—*Ratio of Observed to Expected Number of Cases of Oesophageal Cancer for Combined Habits of Smoking and Drinking*

| | Tobacco smoked | Alcohol None | Alcohol Local | Alcohol Western | Total |
|---|---|---|---|---|---|
| (a) | None | 0·3 | 0·1 | 0·3 | 0·2 |
| Unstandardized | Cigarette tobacco | *1·2* | 0·3 | 0·7 | 0·7 |
| | Pipe tobacco | *1·0* | 1·5 | 1·2 | 1·2 |
| | Cigarette and pipe tobacco | *1·0* | 0·9 | 1·7 | 1·5 |
| | Total | 0·6 | 0·8 | 1·2 | 1·0 |
| (b) | None | 0·3 | 0·1 | 0·3 | 0·3 |
| Standardized | Cigarette tobacco | *1·1* | 0·3 | 0·8 | 0·7 |
| for tribe | Pipe tobacco | *0·8* | 1·3 | 1·1 | 1·1 |
| | Cigarette and pipe tobacco | *1·0* | 1·0 | 1·7 | 1·5 |
| | Total | 0·6 | 0·8 | 1·2 | 1·0 |

TABLE VIII.—*Combined Habits of Smoking and Drinking in Durban Africans, Showing Expected Number of Cases According to Age*

| | Alcohol | | | | | | | | | | | |
|---|---|---|---|---|---|---|---|---|---|---|---|---|
| | None | | | Local | | | Western | | | Total | | |
| Tobacco smoked | Cases | Cont. | Age Exp. | Cases | Cont. | Age Exp. | Cases | Cont. | Age Exp. | Cases | Cont. | Age Exp. |
| None | 5 | 29 | 10·3 | 5 | 38 | 14·4 | 5 | 32 | 11·9 | 15 | 99 | 36·6 |
| Cigarette tobacco | 1 | 9 | 1·8 | 1 | 12 | 3·2 | 3 | 40 | 10·1 | 5 | 61 | 15·1 |
| Pipe tobacco | 2 | 4 | 1·2 | 11 | 43 | 13·3 | 12 | 39 | 12·5 | 25 | 86 | 27·0 |
| Cigarette and pipe tobacco | 2 | 4 | 0·8 | 8 | 12 | 3·1 | 38 | 43 | 10·4 | 48 | 59 | 14·3 |
| Total | 10 | 46 | 14·1 | 25 | 105 | 34·0 | 58 | 154 | 44·9 | 93 | 305 | 93·0 |

TABLE IX.—*Ratio of Observed to Expected Number of Cases of Oesophageal Cancer for Combined Habits of Smoking and Drinking in Durban Africans (Age Adjusted)*

| Tobacco smoked | Alcohol None | Alcohol Local | Alcohol Western | Total |
|---|---|---|---|---|
| None | 0·5 | 0·3 | 0·4 | 0·4 |
| Cigarette tobacco | *0·6* | *0·3* | 0·3 | 0·3 |
| Pipe tobacco | *1·7* | 0·8 | 1·0 | 0·9 |
| Cigarette and pipe tobacco | *2·5* | *2·6* | 3·7 | 3·4 |
| Total | 0·7 | 0·7 | 1·3 | 1·0 |

analysis of smoking and drinking patterns of Durban African males (who are predominantly Zulus) to be made.

Table VIII provides the smoking and drinking patterns of Durban African male oesophageal cancer cases and controls. The third column shows the expected number of cases according to age, as cases and controls were not distributed equally in terms of age (see tribal standardization above).

The ratios of the observed to expected numbers of oesophageal cancer cases in terms of smoking and drinking habits are shown in Table IX, and this Table is comparable with Table VII. Once again, the drinking habit exerts no consistent effect whereas the smoking habit produces higher ratios when pipe tobacco (alone or with cigarettes) is used. These figures are entirely in accord with the Johannesburg analysis.

### DISCUSSION

No idiosyncratic tribal smoking or drinking habits have been found in these urban African groups, but tribal affiliation may influence an individual smoker towards a preference for a certain smoking habit. We think that it is in this way that tribal affiliation has bearing on the development of oesophageal cancer.

This study is to some extent comparable with the work of Schwartz, Denoix and Anguera (1957), Wynder and Bross (1961) and Schoenberg, Bailar and Fraumeni (1971). All these investigations showed an epidemiological association between oesophageal cancer and the consumption of tobacco and alcohol, although Schoenberg *et al.* (1971) considered that a third factor, possibly urbanization, was more important than tobacco or alcohol. Cook (1971) has suggested an association between the occurrence of cancer of the oesophagus in Africa and the use of maize as an ingredient of beer.

The finding of this study is that smoking is a far more powerful oesophageal insult than drinking. Cigarette tobacco does not appear to be a significant oesophageal insult but pipe tobacco does, and the use of both these types may have a synergistic effect. We have been unable to demonstrate that alcoholic oesophageal insults are important in the development of oesophageal cancer in this African male population, and cannot confirm Cook's hypothesis about maize beer or Burrell's suspicion of concoctions.

We would like to thank Professor H. Seftel, Non-European Hospital, Johannesburg, who permitted the collection of data, and Mr C. P. S. Barnard of the Chamber of Mines, who assisted with the recording of data. We are also grateful to Dr J. S. Harington, Cancer Research Unit of the National Cancer Association of South Africa for assistance in the preparation of this paper, which is one of a series initiated by the late Dr A. G. Oettlé that he left uncompleted at the time of his death in 1967.

### REFERENCES

BRADSHAW, E. & SCHONLAND, M. (1969) Oesophageal and Lung Cancers in Natal African Males in Relation to Certain Socio-economic Factors. *Br. J. Cancer*, **23**, 275.

BURRELL, R. J. W. (1957) Oesophageal Cancer in the Bantu. *S. Afr. med. J.*, **32**, 401.

BURRELL, R. J. W. (1962) Esophageal Cancer among Bantu in the Transkei. *J. natn. Cancer Inst.*, **28**, 496.

CLEMMESEN, J. (1965) Statistical Studies in Malignant Neoplasms. *Acta path. microbiol. scand.*, Suppl. 174.

COOK, P. (1971) Cancer of the Oesophagus in Africa: A Summary of the Evidence for the Frequency of Occurrence, and a Preliminary Indication of the Possible Association with the Consumption of Alcoholic Drinks made from Maize. *Br. J. Cancer*, **25**, 853.

HAMMOND, E. C. (1964) Smoking in Relation to Mortality and Morbidity. *J. natn. Cancer Inst.*, **32**, 1161.

HIGGINSON, J. & OETTLÉ, A. G. (1960) Cancer Incidence in the Bantu and "Cape Colored" Races of South Africa: Report of a Cancer Survey in the Transvaal (1953–55). *J. natn. Cancer Inst.*, **24**, 589.

OETTLÉ, A. G. (1967) Cancer Research in Africa, Illustrated by a Recent Epidemic of Cancer of the Gullet. *Raymond Dart Lectures No.* 3. Witwatersrand University Press (for the Institute for the Study of Man in Africa).

ROBERTSON, M. A., HARINGTON, J. S. & BRADSHAW, E. (1971) The Cancer Pattern in Africans at Baragwanath Hospital, Johannesburg. *Br. J. Cancer*, **25**, 377.

SCHOENBERG, B. S., BAILAR, J. C. & FRAUMENI, J. F. (1971) Certain Mortality Patterns of Esophageal Cancer in the United States, 1930–67. *J. natn. Cancer Inst.*, **46**, 63.

SCHONLAND, M. & BRADSHAW, E. (1968) Cancer in the Natal African and Indian, 1964–66. *Int. J. Cancer*, **3**, 304.

SCHWARTZ, D., DENOIX, P. F. & ANGUERA, G. (1957) Recherche des localisations du cancer associées aux facteurs tabac et alcool chez l'homme. *Bull. Ass. fr. étude Cancer*, **44**, 336.

SCHWARTZ, D. R., FLAMANT, R., LELLOUCH, J. & DENOIX, P. F. (1961) Results of a French Survey on the Role of Tobacco. *J. natn. Cancer Inst.*, **26**, 1085.

SKINNER, M. E. G. (1967) Malignant Disease of the Gastro-intestinal Tract in the Rhodesian African, with Special Reference to the Urban Population of Bulawayo: a Preliminary Report. *Natn. Cancer Inst. Monog.* No. 25, 57.

STASZEWSKI, J. (1960) Statistical Data on Smoking and "Tobacco-Tract" Cancer in Poland. *Br. J. Cancer*, **14**, 419.

WYNDER, E. L. & BROSS, I. J. (1961) A Study of Etiological Factors in Cancer of the Esophagus. *Cancer, N.Y.*, **14**, 389.